# Shattering the Water Window: Comprehensive Mapping of Faradaic Reactions on Bioelectronics Electrodes

**DOI:** 10.1021/acsami.4c12268

**Published:** 2024-10-01

**Authors:** Jiří Ehlich, Čeněk Vašíček, Jan Dobeš, Amedeo Ruggiero, Markéta Vejvodová, Eric Daniel Głowacki

**Affiliations:** † Bioelectronics Materials and Devices Laboratory, Central European Institute of Technology CEITEC, Brno University of Technology, Purkyňova 123, Brno 61200, Czech Republic; ‡ Department of Chemistry, Faculty of Science, Masaryk University, Kotlářská 2, Brno 611 37, Czech Republic

**Keywords:** electrochemistry, neurostimulation, platinum electrodes, bioelectronics, reactive oxygen species, reactive chlorine species, water window

## Abstract

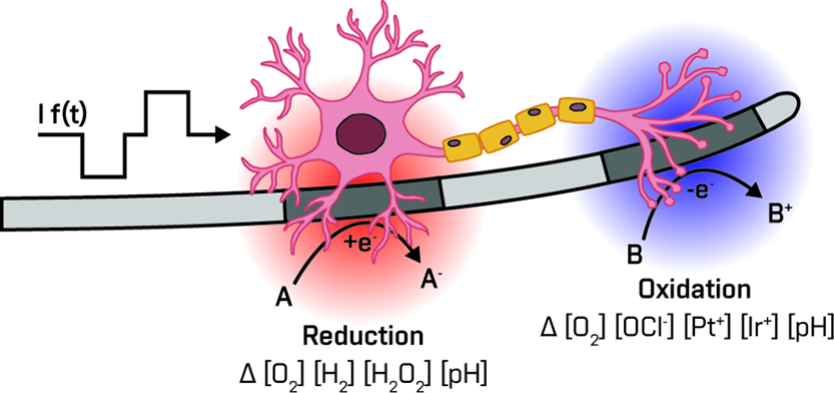

It is generally accepted that for safe use of neural interface electrodes, irreversible faradaic reactions should be avoided in favor of capacitive charge injection. However, in some cases, faradaic reactions can be desirable for controlling specific (electro)physiological outcomes or for biosensing purposes. This study aims to systematically map the basic faradaic reactions occurring at bioelectronic electrode interfaces. We analyze archetypical platinum–iridium (PtIr), the most commonly used electrode material in biomedical implants. By providing a detailed guide to these reactions and the factors that influence them, we offer a valuable resource for researchers seeking to suppress or exploit faradaic reactions in various electrode materials. We employed a combination of electrochemical techniques and direct quantification methods, including amperometric, potentiometric, and spectrophotometric assays, to measure O_2_, H_2_, pH, H_2_O_2_, Cl_2_/OCl^–^, and soluble platinum and iridium ions. We compared phosphate-buffered saline (PBS) with an unbuffered electrolyte and complex cell culture media containing proteins. Our results reveal that the “water window”—the potential range without significant water electrolysis—varies depending on the electrolyte used. In the culture medium that is rich with redox-active species, a window of potentials where no faradaic process occurs essentially does not exist. Under cathodic polarizations, significant pH increases (alkalization) were observed, while anodic water splitting competes with other processes in media, preventing prevalent acidification. We quantified the oxygen reduction reaction and accumulation of H_2_O_2_ as a byproduct. PtIr efficiently deoxygenates the electrolyte under low cathodic polarizations, generating local hypoxia. Under anodic polarizations, chloride oxidation competes with oxygen evolution, producing relatively high and cytotoxic concentrations of hypochlorite (OCl^–^) under certain conditions. These oxidative processes occur alongside PtIr dissolution through the formation of soluble salts. Our findings indicate that the conventional understanding of the water window is an oversimplification. Important faradaic reactions, such as oxygen reduction and chloride oxidation, occur within or near the edges of the water window. Furthermore, the definition of the water window significantly depends on the electrolyte composition, with PBS yielding different results compared with culture media.

## 1 Introduction

The fields of bioelectronics and neural engineering have witnessed significant advancements in recent years. Electronic devices, exploiting electrode interfaces with biological systems, are used in many fields of basic biophysical research as well as neurostimulation in animals and humans.^1−4^ Neural stimulation technologies play a pivotal role in various medical devices and bioelectronic medicine. These technologies have found applications in cardiac pacemakers, cochlear implants, deep brain stimulators, and peripheral nerve stimulators, among others.^5^ Central to the effectiveness and safety of these neuromodulation devices are the electrochemical properties of the materials used in their construction. Understanding the electrochemical processes that occur at the electrode–tissue interface during neural stimulation is critical for optimizing device performance and ensuring patient safety.^9−11^ Previous research has delved into the electrochemistry of noble metals like platinum (Pt) and PtIr alloys, shedding light on the capacitive, faradic and pseudofaradaic charge injection mechanisms, and their characterization protocols.^12^ Brummer and Turner published a series of classic studies on platinum charge injection electrochemistry for neurostimulation in the 1970s, outlining and quantifying several key aspects to faradaic reactions.^13−18^ Comprehensive exploration of the mechanisms on Pt/PtIr and other electrodes during neural stimulation nevertheless remains an area of active investigation, and unanswered questions remain. This work aims to supplement these earlier studies and fill in some missing pieces. Aside from questions of safety or spurious effects of neurostimulation, which are dominant in the neural interface field, the same questions about electrochemical reactions are increasingly relevant across different disciplines of bioelectronics: from biophysical experiments on cells to electrochemical cancer treatments and biosensors. There are examples of targeted use of faradaic processes (with direct current) to trigger a given biological outcome,^19−21^ yet systematic mapping of reactions in biologically relevant media, even on Pt, is lacking.

In this article, we present a study focused on the direct mapping of faradaic reactions in biological media. The goal is to create an experimental framework that can be used to test any electrode material. As an archetypical electrode, we test PtIr, in the form of a clinically approved electrode system, namely, stereoencephelography (SEEG) electrodes. PtIr (typically 10–15% Iridium content) is by far the most widely used electrode material in clinically approved implantable devices.^6−8^ It should be noted that such Pt alloys behave electrochemically like pure Pt and do not show characteristics of Ir electrochemistry. Though we used PtIr in this work, the methods we applied are generally applicable to other electrode materials.

**Figure 1:**
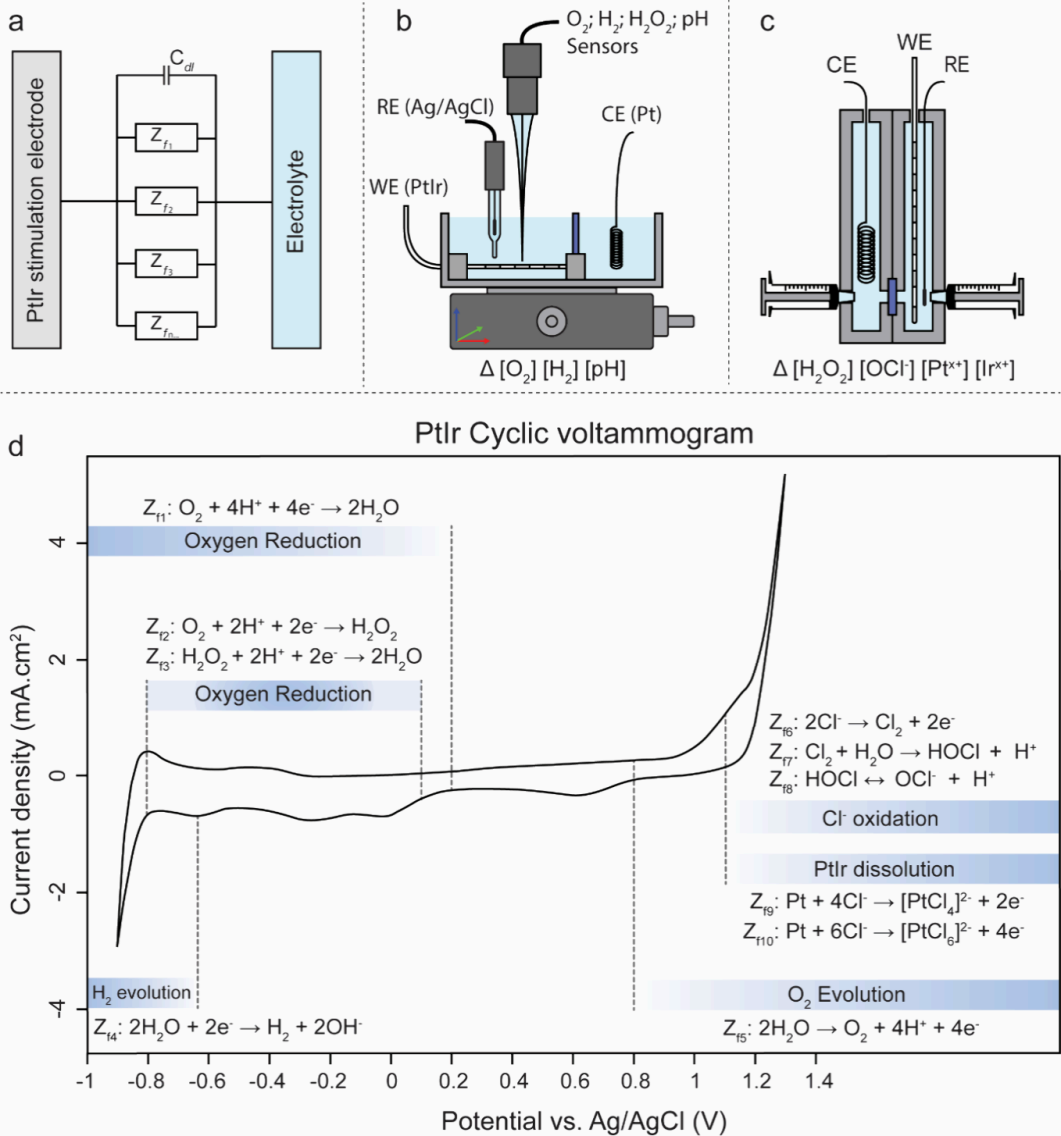
Overview of the study and key results. (a) Equivalent circuit diagram of an electrode–electrolyte interface, illustrating capacitive and faradaic routes of charge transfer. (b) Experimental setup for assessing oxygen reduction/evolution, hydrogen peroxide evolution, hydrogen evolution, and pH changes at a PtIr electrode interface. In this configuration, the potential of the electrode under test is controlled in a three-electrode configuration with a potentiostat, and changes in the species of interest are recorded by matching amperometric/potentiometric microsensors, which can be recorded in the spatial vicinity of the PtIr electrode surface. (c) Experimental H-cell setup for quantification of the generation of hydrogen peroxide, hypochlorite, and PtIr dissolution products. This setup provides a “bulk” electrolysis to accumulate the product, which is then quantified by a spectrophotometric or mass spectroscopy method. (d) Master cyclic voltammogram (CV) plot showing a conventional CV trace of a PtIr electrode in PBS under ambient air conditions. The given onset potentials of the faradaic reactions were determined according to tracking of reaction products, as shown in panels (b) and (c).

Our investigation involved the mapping of various faradaic reactions that may transpire during neural stimulation (Figure 1a), schematized as reactions Z_f1_–Z_f10_ by combining electrochemical experiments with *in situ* measurements of possible electrochemical products. We began by examining oxygen reduction reactions (Z_f1_–Z_f3_), which play a significant role during the cathodic charge injection phase used in neurostimulation. To assess these reactions, we measured oxygen depletion near the PtIr electrode by using an amperometric microsensor (Figure 1b). The evolution of hydrogen peroxide was measured in two ways: (1) characterizing local concentration, using an amperometric probe, according to our previously published method,^22^ and (2) average concentration, using a custom-made H-cell setup and spectrophotometric assay for quantification (Figure 1c). These investigations allowed us to gain a comprehensive understanding of the electrochemical pathways involved in oxygen reduction on PtIr electrodes and the dynamics of peroxide production/decomposition. Building on this foundation, we shifted our focus to water electrolysis, a process known to influence the surrounding environment through oxygen evolution, hydrogen evolution, and pH changes (Z_f4_–Z_f5_). To accomplish this, we employed amperometric microsensors and a micro-pH electrode positioned in close proximity to the electrode (b).

Another crucial question was the investigation of chloride ion oxidation on PtIr (Z_f6_–Z_f8_), with a specific focus on the production of hypochlorite (OCl^–^). Hypochlorite is a very strong oxidizing agent used in rocket propellants, bleaching agents, disinfectants, and herbicides. While previous studies often emphasized platinum dissolution in the presence of chloride ions, the generation of hypochlorite, which is actually produced by electrolysis on the industrial scale,^23−25^ received limited attention. There are references mentioning hypochlorite evolution as a possibility during electrical stimulation; however, to the best of our knowledge, this has not been measured to date.^13,16^ Our work bridges this gap by providing quantitative measurements of hypochlorite production, shedding light on this often-overlooked reaction. To finalize the study, we have also measured platinum and iridium dissolution (Z_f9_–Z_f10_), which may lead to solubilized metal salts, including the accumulation of the strong oxidizing agent [PtCl_6_]^2–^ known for its toxicity to cells.

Our study was conducted in two distinct electrolytes: standard phosphate-buffered saline (PBS) solution, serving as a simple model buffered electrolyte containing only inorganic ions, and Dulbecco’s Modified Eagle Medium (DMEM) supplemented with Fetal Bovine Serum (FBS). The choice of DMEM with FBS was motivated by observations suggesting that electrochemical reactions might occur under different potentials in more complex physiological media than in simple PBS. The presence of proteins, which can adsorb to electrodes, can cause changes in electrochemical processes.^18^ Moreover, culture media are rich with antioxidant compounds, which may be readily consumed during application of anodic potentials.^13^ Thus, PBS serves as a “clean” buffered reference, while DMEM+FBS represents a scenario rich with organic redox substrates. We have also added unbuffered electrolytes like sodium sulfate to serve as comparisons for pH change effects in nonbuffered electrolytes. For experiments on the positive identification of chloride oxidation, we compared chloride-containing vs chloride-free electrolytes.

In summary, this work represents an advancement in our understanding of faradaic reactions on PtIr neuromodulation electrodes. By mapping these reactions, we have unveiled the potentials at which they occur and quantified their extent in terms of the concentration. We have measured oxygen evolution/depletion, hydrogen evolution, and accompanying pH changes. We have described the accumulation of strong oxidizing reagents hydrogen peroxide, hypochlorite, and solubilized platinum/iridium ions, which might be, together with the pH changes and localized hypoxic conditions, the reason behind adverse effects of electrical stimulation. This knowledge serves as a critical foundation for our future investigations, where we aim to explore these reactions under various charge-balanced biphasic stimulation protocols. Such research will help us assess the occurrence and accumulation of faradaic byproducts, contributing to the development of safer neuromodulation protocols and potentially opening doors to novel applications of these electrochemical reactions in neural engineering and bioelectronic devices more broadly.

## 2 Experimental Methods

We employed commercial DIXI Medical MICRODEEP Stereoelectroencephalography (SEEG) electrodes in our study. These electrodes consist of 18 independently addressable cylindrical segments, each with a diameter of 0.8 mm and a length of 2 mm. They are constructed from a platinum–iridium alloy in a 90:10 ratio.

### 2.1 Electrochemical Cells

Two custom electrochemical cells were purpose-built for our experiments. The first cell (see Figure 1b) was designed for precise positioning of amperometric and potentiometric microsensors above the electrode under investigation. This 40 mL cell was crafted from a high-density polyethylene block using our in-house CNC router. The front window consisted of a microscope glass slide, secured with 3D-printed polypropylene clamps, and sealed with PDMS (Sylgard 184) to prevent electrolyte leakage. The window allowed for visual guidance during electrode positioning, facilitated by a digital microscope (Q-SCOPE 20200-P). Accurate electrode placement was achieved using a micrometer-precision XYZ-stage from ThorLabs. The SEEG electrode was inserted through a hole on the side of the cell and secured horizontally with 3D-printed polypropylene clamps. This insertion hole was sealed with PDMS. A platinum wire coil with a surface area of approximately 7 cm^2^ served as the counter electrode, and a standard silver/silver chloride reference electrode with a 3 M KCl filling solution was employed (Redox.me). The counter electrode was separated from the main chamber by a 3D-printed salt bridge, consisting of a porous poly(lactic acid) plug, which impedes diffusion of electrochemical products between the working and counter electrode chambers.

The second cell, which we will refer to as the H-cell (see Figure 1c), was utilized for spectroscopic measurements and constructed from clear polycarbonate sheets shaped by a CNC router. Individual components were assembled with screws and rendered leak-proof with PDMS. This cell featured two compartments separated by a salt bridge composed of a 0.007 in thick Nafion 117 membrane (Sigma-Aldrich). Each compartment had a 1 mL volume and could be easily filled or emptied using syringes press-fitted on the side of the cell. A pseudoreference electrode made from anodized silver wire (+100 mV in PBS vs standard silver/silver chloride) was placed in the compartment with the SEEG electrode, while a platinum counter electrode with a surface area of approximately 20 cm^2^ was positioned in the second compartment. For the quantification of dissolved platinum and iridium, a similar cell was constructed. The only difference was the working electrode chamber consisted of a glass tube to prevent adsorption of the PtIr dissolution products and easy cleaning of the chamber with Aqua regia.

### 2.2 Electrochemical Measurements

All electrode potentials given in this work are versus a Ag/AgCl reference electrode in 3 M KCl, unless otherwise noted. Chronoamperometric measurements and cyclic voltammetry in the cell with amperometric sensors were conducted using the Ivium Vertex.s 5A potentiostat. Amperometric measurements for spectroscopic analysis in the second cell were performed with an Ivium PocketSTAT2 potentiostat. Three different electrolytes were used across the different electrochemical measurements: standard 0.1 M phosphate-buffered saline (PBS) was utilized as a reference electrolyte, commonly employed for the *in vitro* characterization of electrodes intended for neuromodulation applications. We prepared this PBS using ROTIFair PBS 7.4 tablets. To replicate a complex *in vitro* condition, we employed Dulbecco’s Modified Eagle Medium (DMEM) buffered with 25 mM of 4-(2-hydroxyethyl)-1-piperazineethanesulfonic acid (HEPES) from Gibco, ThermoFisher Scientific (21063-029), supplemented with 10% Fetal Bovine Serum, not deactivated, FBS also from Gibco, ThermoFisher Scientific (A3160801), and 1% PenStrep also from Gibco (15140148) as an electrolyte. To emphasize observed pH changes or eliminate any contribution from chloride ions to oxidative currents, we used pure 0.1 M sodium sulfate solution as a reference electrolyte. This sodium sulfate solution does not possess buffering capacity and is free of chloride ions. In some tests, we also employed a chloride-free phosphate buffer solution to resolve the onset of chloride oxidation versus water oxidation, but with preserving buffering capacity.

### 2.3 Microsensors

We assessed local gradients in dissolved oxygen, hydrogen, and pH near the electrode using a commercial microprobe system provided by *Unisense A/S*, Denmark. The system comprised a UniAmp multichannel amplifier, SensorTrace Logger software, and analyte-specific microsensors. The microsensors employed in our measurements were as follows:

- An OX-50 amperometric microsensor with a 50 μm diameter tip for oxygen detection.
- An H2-50 amperometric microsensor with a 50 μm diameter tip for hydrogen detection.
- A PH-200 potentiometric microsensor with a 200 μm diameter tip for pH quantification.
- A World Precision Instruments (WPI) ISO-HPO-2 hydrogen peroxide sensor, with a 1 mm diameter sensor tip. Here, the WPI free-radical analyzer amplifier system was used, with data collected using the Lab-Trax4/16 (WPI) with LabScribe software.

The amperometric oxygen sensor measured the cathodic current on the polarized working electrode inside the sensor, resulting from the reduction of oxygen passing through a polymeric gas-permeable membrane. The resulting current was directly proportional to the partial oxygen pressure in the sensor environment. The hydrogen sensor operated on a similar principle, with hydrogen being oxidized on the sensor’s working electrode. The pH sensor functioned as a standard pH electrode, where the pH was determined based on the potential difference between the sensing glass electrode and a distant standard Ag/AgCl reference electrode. In the case of the PH-200 microsensor, these electrodes were separate units. The glass microelectrode recorded the local potential versus the distant standard Ag/AgCl reference electrode. All microsensors were used in accordance with the manufacturer’s instructions and calibrated before each measurement. The peroxide sensor works on a similar principle, amperometrically detecting oxidation of peroxide, which permeates through a protective membrane. It should be noted that this sensor is cross-sensitive with H_2_ and thus should only be used to quantify peroxide in situations where there is certainty that H_2_ is not being evolved as well.

### 2.4 Determination of Hydrogen Peroxide and Hypochlorite

We quantified hydrogen peroxide generated during the experiments using the amperometric method described above and also using a spectrophotometric assay and the H-cell configuration. This assay relied on the oxidation of 3,3′,5,5′-tetramethylbenzidine (TMB) in the presence of horseradish peroxidase (HRP) and citric acid–phosphate buffer solutions.^26^ Absorbance values were measured at a wavelength of 653 nm using a Biotek Synergy H1 microplate reader spectrometer. Depending on the concentration of hydrogen peroxide in the sample, different aliquot volumes were taken and added to the corresponding volumes of HRP/TMB/buffer solution. Concentration values were determined based on calibration curves, which were regularly obtained before measurements. Hypochlorite was also quantified through a spectrophotometric assay based on the oxidation of TMB,^27^ which is accompanied by a change of absorbance at the 653 nm wavelength; in this case, there is no need to add HRP as hypochlorite reaction with TMB is spontaneous and fast. First, a TMB calibration plot had to be obtained. Commercial Ca(ClO)_2_ was dissolved to prepare a stock hypochlorite solution that was standardized by the iodometry technique, and then it was used to prepare known OCl^–^ solutions by subsequent dilution. TMB solution mixed with different amounts of OCl^–^ showed a linear response to increasing concentrations, up to 80 μM, allowing the building of a calibration curve to use in hypochlorite quantification experiments. Using such a calibration curve, the hypochlorite content in the sample was obtained by mixing it with TMB, measuring the absorbance and extrapolating the concentration.

### 2.5 Determination of Dissolved Pt and Ir

PtIr electrodes were pretreated by reduction and CV cycling to create a clean surface as a standard starting condition (−1 mA·cm^–2^ applied for 30 min, followed by 100 CV scans −800 mV to +1 V) in PBS, followed by rinse in DI water. The anodic corrosion experiment was carried out on this precleaned electrode inside of the H-cell setup. Stepwise anodic chronoamperometry was conducted in the range from −0.1 to 2 V. Each anodic potential step, starting at 0 V, was applied for 30 min. After each potential, the anode chamber electrolyte was collected for analysis, and the whole cell was rinsed with 5% aqua regia and DI to remove any Pt and Ir contamination and reset the setup before the application of the next highest potential. The concentrations of ^195^Pt and ^193^Ir were determined by inductively coupled plasma mass spectrometry, ICP-MS, using an Agilent7900 ICP-MS with a MicroMist nebulizer, Scott-type double-pass spray chamber, and SPS 4 Autosampler device. To reach the maximum sensitivity, the ICP-MS instrument was tuned using a solution of ^193^Ir, ^195^Pt, and ^205^Tl at 0.5 μg L^–1^ each. A collision–reaction cell with helium was used to reduce possible interferences.

All samples were diluted with 2% HCl based on matrix concentration to reduce plasma quenching by organic substances present. The calibration set was prepared using Astasol (Analytika, s.r.o.) certified reference materials of Ir, Pt, and Tl with original concentration 1000 ± 2 mg L^–1^, pure media, (PBS, DMEM) and 2% HCl for matrix-matched calibration.

The measurement protocol consisted of properly washing tubing with 2% HCl (analpure) and 2% HNO_3_ (analpure), rinsing the autosampler probe, taking up time, stabilizing time, and measuring (data acquisition). The peak measurement protocol was based on measuring 3 points per peak with 5 replicates and 50 sweeps per replicate. MassHunter software was used for data collection and evaluation. Where necessary, the software automatically performed internal standard correction (Tl).

## 3 Results and Discussion

### 3.1 Cyclic Voltammetry Characterization

**Figure 2:**
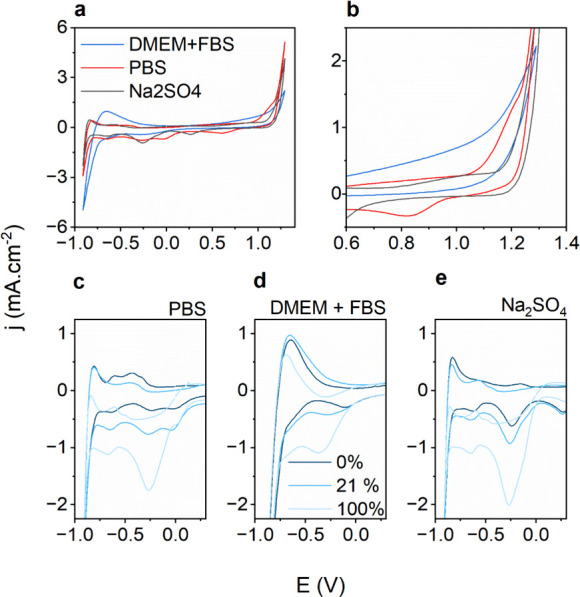
(a) Cyclic voltammetry of PtIr electrodes in three different electrolytes: PBS, DMEM+FBS, and phosphate-buffered Na_2_SO_4_ (chloride-free reference). Conditions: 21% oxygen saturation, scan rate = 100 mV s^–1^, the 10th scan cycle is shown in each panel. (b) Detail of the same CVs zoomed on the anodic oxidation, including chloride-free phosphate buffer for comparison to reveal the onset of the chloride oxidation and chlorine rereduction reactions. (c–e) CVs focus on the cathodic oxygen reduction and hydrogen evolution reactions for all three electrolytes in 100% oxygenated (pale blue), 21% oxygenated (ambient air, blue), and 0% oxygenated (100% N_2_ purged, dark blue) conditions.

A starting point for understanding the electrochemical reactions occurring at PtIr is cyclic voltammetry (CV, Figure 2a). This is the most popular characterization method, which can provide important clues about the occurrence of different reactions. The characteristic “textbook” peaks on Pt or PtIr have been described in the literature in considerable detail;^28−30^ thus, we will focus on the interpretation of CVs in the context of the specific reactions we later quantify in this work. A lot can be learned about what reactions are occurring by comparing different electrolytes with certain reagents added or subtracted. In considering anodic polarizations (Figure 2b), with comparing PBS and Na_2_SO_4_ electrolytes, it is reasonable to assume that, by 1.2 V, the same oxidation process is occurring in both electrolytes, and that process must be OER. In PBS, the anodic current is larger than in Na_2_SO_4_ and has an earlier onset potential. Comparing PBS with Cl-free phosphate buffer indicates where this anodic current originates from chloride oxidation. This chloride oxidation is accompanied by a characteristic rereduction cathodic peak at around +800 mV. Therefore, it can be said that in PBS, chloride oxidation occurs in a similar potential range as the OER and these processes may coexist. In later sections, we show how measurement of O_2_ and HOCl concentrations will provide a definitive assignment of which reactions occur at which potentials. The anodic CV in DMEM+FBS reveals substantially higher anodic current overall until the OER potential is reached, with markedly higher anodic currents even at potentials around +100 mV. This indicates that there are one or more species in the medium that can be oxidized at potentials lower than those necessary to drive the OER and chloride oxidation. The medium contains easily oxidizable organic content, such as glucose, various proteins, and other antioxidants, and therefore, many oxidation processes are available.^13^ It is not clear which specific compounds are being oxidized; however, biological media are rich with antioxidants. Meanwhile, at higher potentials corresponding to the OER, currents are higher in PBS versus DMEM+FBS, most likely due to the presence of biofouling in the medium, which impedes the maximum OER current. Cathodic polarizations on the CV plot (Figure 2a) reveal regions of ORR, between roughly 0 and −0.6 V, and then prominent HER at more negative voltages. Backward scans reveal characteristic hydrogen reoxidation peaks (around −900 mV) and hydride desorption peaks.^28^ To clearly resolve if ORR is occurring, one can prepare electrolytes with 0% oxygen (bubbled with N_2_), 21% oxygen (ambient conditions), or 100% oxygen, with bubbling of pure O_2_ (Figure 2 c,d,e). In comparing these conditions of oxygenation, at negative potentials of −600 mV or more, HER is clearly dominant, while ORR occurs at less cathodic potentials, apparently already from 0 V. An interesting observation is that in DMEM+FBS, there is a sharp increase in cathodic current at less negative potentials than in the other electrolytes. The process could be some organic molecule reduction, or HER. From interpreting CV alone, it is difficult to discriminate reactions other than HER; however, later amperometric measurements will confirm that indeed the onset of HER is at lower overpotentials in the DMEM medium than in the other electrolytes.

### 3.2 Oxygen Reduction Reactions (ORR): Oxygen Depletion and Peroxide Formation

**Figure 3:**
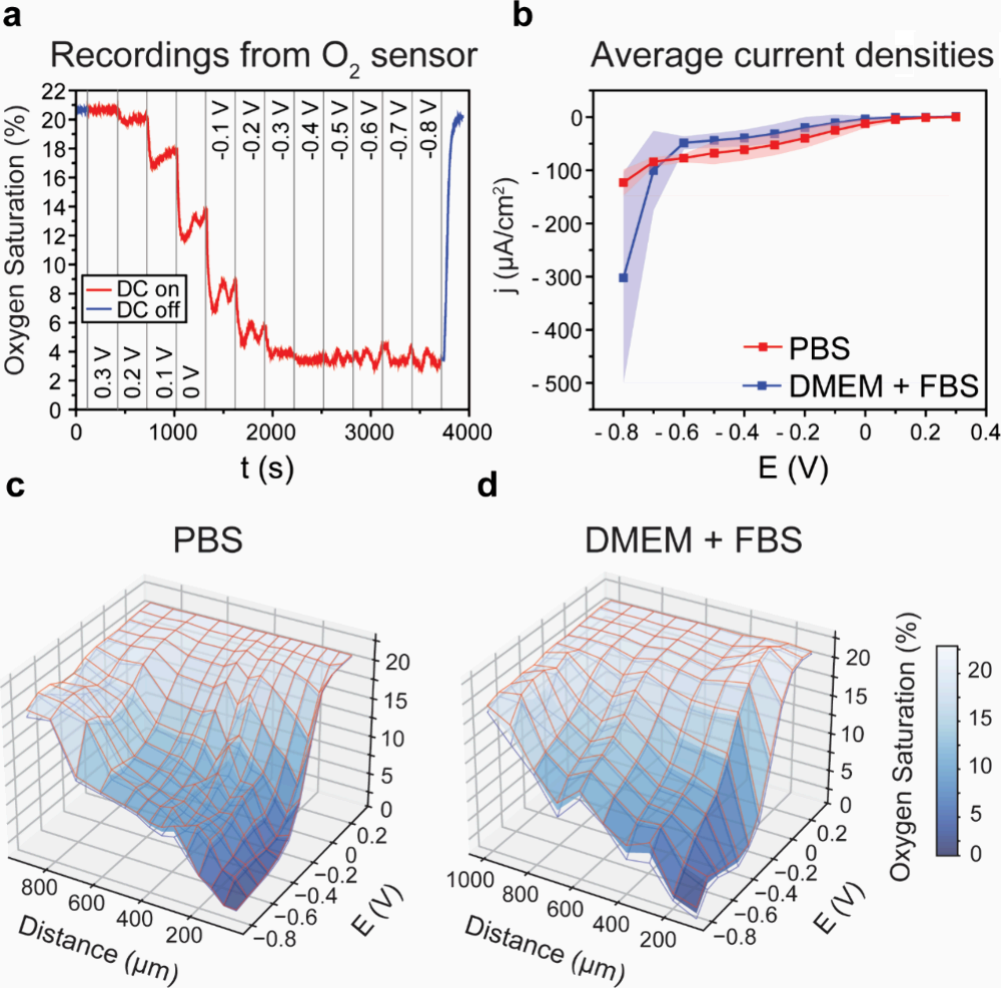
Oxygen reduction reaction (ORR) and the effect of oxygen depletion at PtIr electrodes. (a) Chronoamperometric voltage-step experiment from +300 to −800 mV, holding each potential for 5 min and registering the oxygen concentration [O_2_] measured at a distance of 100 μm above the PtIr electrode in PBS. The blue part of the trace signifies when the DC is off; the red part shows where it is turned on. After application of −800 mV, the DC is turned off and oxygen rapidly diffuses to nullify the gradient. (b) Equilibrium current density values recorded during chronoamperometry; these currents correspond to the diffusion-limited ORR process, until −800 mV where H_2_ evolution takes over. Average current from three measurements ± SD. Once HER dominates, hydrogen bubble formation on the electrode surface causes higher dispersion in the measured current values. (c) 3D plot of the oxygen depletion gradient formation effect as a function of distance from the PtIr electrode surface and applied potential, recorded in the PBS electrolyte (c) and in DMEM+FBS (d). The value of 21% represents O_2_ saturation under ambient air.

From CV experiments performed in oxygenated and deoxygenated conditions, it is clear that ORR is a dominant reduction process in the cathodic range down to roughly −600 mV, where HER begins to take over. To confirm and quantify the ORR, we used Clark-type microamperometric sensors to record the dissolved oxygen concentration in the vicinity of the PtIr electrode, while we applied potential steps to the PtIr. The occurrence of ORR would correspond to consumption of dissolved oxygen and a measurable drop in [O_2_]. We performed chronoamperometry on PtIr, starting at +300 mV, and measuring current and local [O_2_] for 5 min before stepping the potential in the cathodic direction by 100 mV steps (Figure 3a, shown for PBS). ORR is already detectable, via a measurable drop in [O_2_], at +200 mV. As the potential is progressively decreased, [O_2_] drops further, and by −400 mV, the [O_2_] reaches its lowest level of roughly 3%. Chronoamperometry allows for determination of the steady-state ORR current densities supported on PtIr (Figure 3b). ORR is a diffusion-limited reaction, and at equilibrium, an [O_2_] concentration gradient will exist, with [O_2_] being depleted at the electrode surface, while 21% remains at the electrolyte/air interface. Using the microamperometric sensor, it is possible to map this [O_2_] gradient as a function of position and applied cathodic potential (Figure 3c,d). The 3D plots shown in Figure 3c,d reveal that a hypoxic gradient can be formed near the electrode surface when a constant cathodic potential. In comparing this effect of electrochemical oxygen depletion in PBS versus DMEM medium, there is little quantitative or qualitative difference, except that HER begins to take over at −800 mV in DMEM, while in PBS, HER is not dominant at this potential. It can be said that in all electrolytes, application of cathodic potentials in the ORR region leads to hypoxic conditions in the vicinity of the electrode.

**Figure 4:**
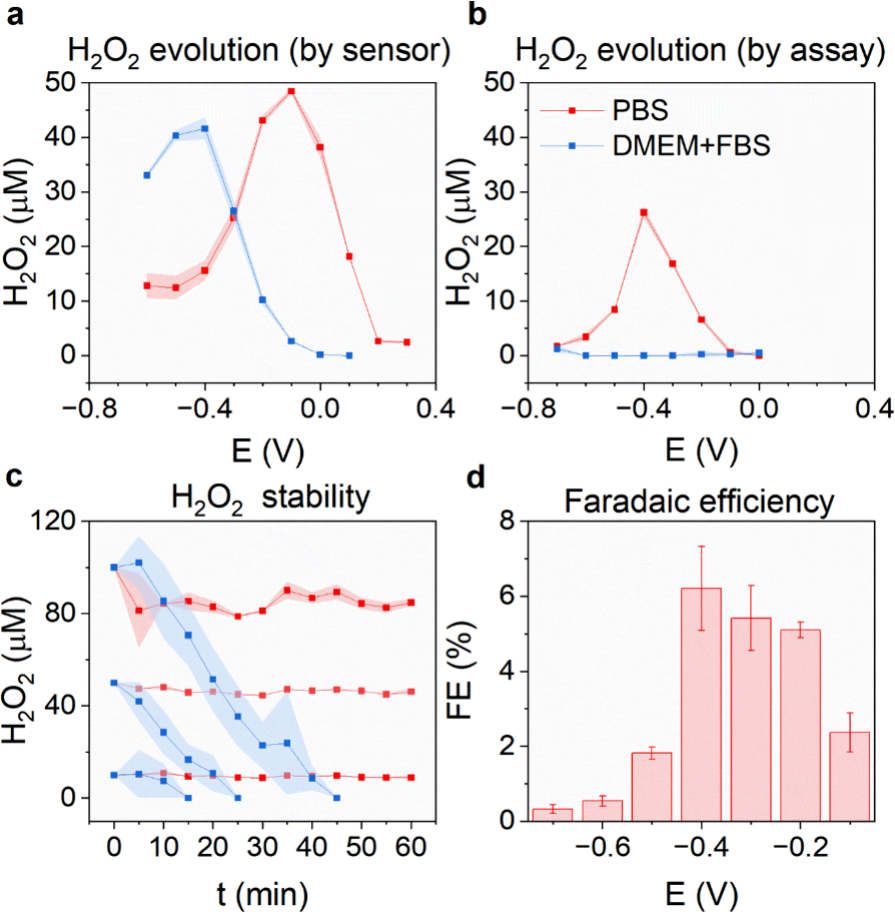
Hydrogen peroxide generation via ORR. (a) Hydrogen peroxide concentration measured locally at a distance of 100 μm above the PtIr electrode surface using an amperometric sensor. The concentration is registered after 5 min of application of a fixed potential. (b) Hydrogen peroxide bulk concentration measured spectrophotometrically after chronoamperometry at a fixed potential for 150 min, red trace = PBS, blue trace = DMEM+FBS. (c) Measurement of the stability of hydrogen peroxide in PBS versus DMEM+FBS upon addition of 100, 50, or 10 μM peroxide in the respective electrolyte. Average concentration from *N* = 3 experimental repetitions ± SD. (d) Faradaic efficiency calculated from total peroxide detected in PBS from *N* = 3 ± SD measurements shown in panel (b).

As indicated in Figure 1, ORR may proceed to water as a product or may generate hydrogen peroxide as an intermediate. This peroxide can accumulate, as shown in our earlier work, the prevalence of peroxide formation depends heavily on the electrode material used.^22,31^ Probing peroxide evolution using local amperometric sensing versus quantification of total concentration spectrophotometrically gives quite different results and insights. The local sensor characterization, measured as a function of voltage applied to the PtIr electrode, reveals a clear peak in peroxide concentration dependent on the applied potential. The peak concentration measured in PBS is found at −100 mV, while in the DMEM+FBS medium, the peak is shifted to −400 mV, with overall less peroxide detected (Figure 4a). At more negative potentials where ORR is occurring, presumably, peroxide is further reduced to water,^22^ limiting its concentration. We conducted chronoamperometry experiments in the H-cell shown in Figure 1c and probed the electrolyte for hydrogen peroxide content (Figure 4b). We found a clear peak of peroxide production at −400 mV in PBS, thus shifted with respect to the peak seen in sensor measurements. The total concentration was roughly half that measured with the sensor. Moreover, in DMEM, only trace amounts of peroxide were detected. To explain these discrepancies, we consider the instability of peroxide, in that it can react with organic substrates in the medium, as well as be reduced or catalytically decomposed in contact with the PtIr electrode surface. This explains why in the medium, the local sensor measurement reveals peroxide formation, while when one measures the total concentration in solution, the peroxide has been consumed by rapidly reacting with available organic molecules in the medium. This will lead to a different outcome for establishing peroxide generation as well as the onset potential. To test this, we conducted a hydrogen peroxide stability/breakdown test, where concentrations of peroxide were pipetted into the electrolyte and measured over time (Figure 4c). While in PBS, the peroxide concentrations remained stable over 60 min, in the medium, we observed a rapid decline. Therefore, it is clear that peroxide reacts with the DMEM medium and is thus consumed in the process. The reaction is a combination of oxidation with organic compounds as well as peroxide decomposition by remaining peroxidase enzymes present in the FBS fraction. Overall, this effect of medium+FBS is an important consideration that though difficult to detect, peroxide may form and rapidly react in biological media, which certainly can have downstream effects that should be taken into account. Overall, the faradaic efficiency of ORR to peroxide on PtIr is low (Figure 4d), considerably lower than other electrode materials like Au^22^ or conducting polymers like PEDOT.^22,32^ The comparison of the sensor vs “bulk” measurement using an optical assay is instructive, and it should be noted that for accurate determination of the onset potential for peroxide generation, the sensor method will lead to more reliable results.

### 3.3 Water Window—H_2_ and O_2_ Evolution and Associated pH Changes

**Figure 5:**
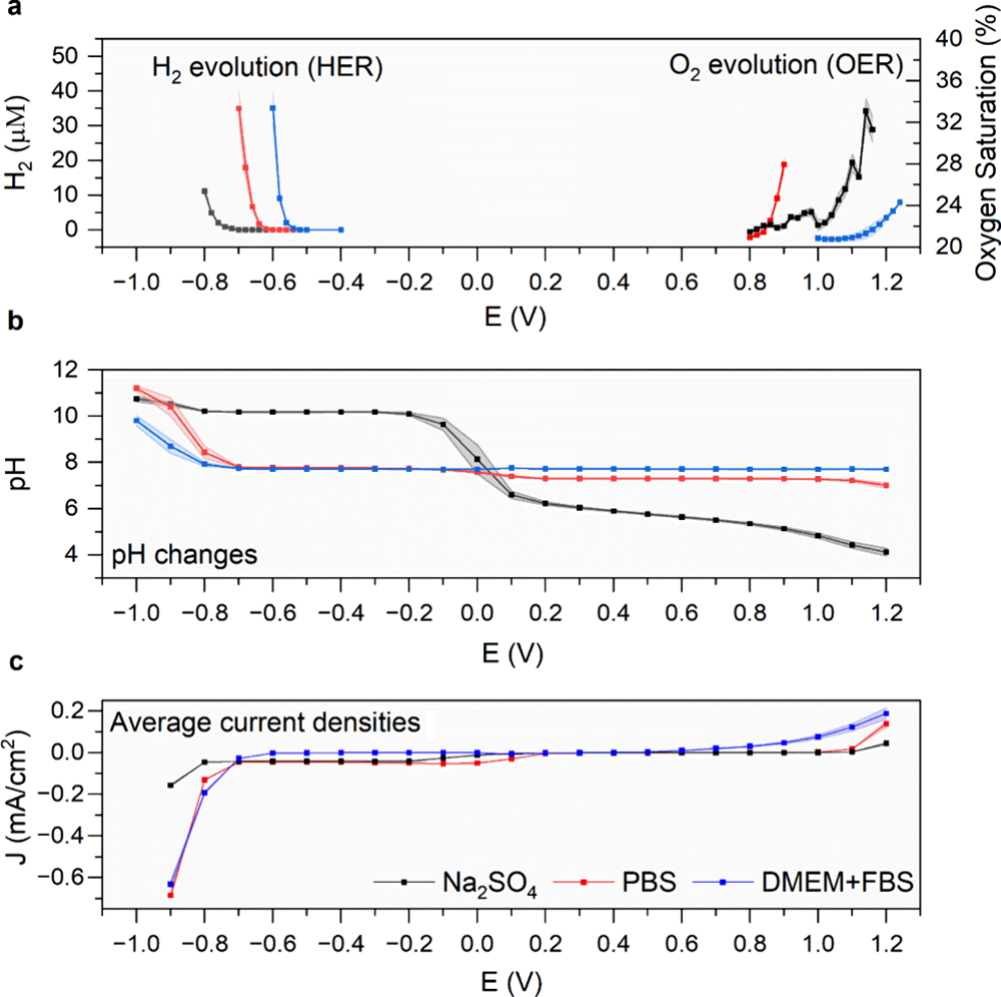
Water electrolysis and the “water window” evaluated using [H_2_], pH, and [O_2_] local measurements. In all cases, the sensor is placed 100 μm above the PtIr electrodes, and potential steps are applied for 5 min. (a) HER tracked by measuring dissolved hydrogen; increase in [O_2_] to track the OER. (b) Changes in pH over the same type of chronoamperometric experiment. Relatively low current densities associated with ORR result in measurable pH changes in unbuffered sodium sulfate, while pH changes in buffered systems are only evident with current densities associated with water electrolysis. (c) Average current density over the 5 min chronoamperometric experiment corresponding to the experiments shown above in panels (a) and (b).

Water-splitting reactions of cathodic HER or anodic OER can be tracked by local measurement of changes in dissolved [H_2_] and [O_2_], respectively. The measurement of H_2_ or O_2_ with local sensors allows for unambiguous assignment of onset potentials for HER and OER. Quantifying local pH changes likewise can be correlated to each reaction. Using the H_2_ sensor, we can precisely assign a cathodic water-splitting onset potential in PBS of −650 mV (Figure 5a). In unbuffered sodium sulfate, by way of comparison, the onset is shifted to −750 mV. This is because of alkalization in unbuffered electrolytes, acting to shift the thermodynamic potential for HER as pH increases.^33^ In the DMEM+FBS medium, on the other hand, we observe a positive shift in the HER onset potential to a value of −550 mV. The pH of the medium is identical to PBS, and in fact, as is apparent in the pH measurements in Figure 5b, the medium is more resistant to pH changes than PBS itself. If pH differences are not responsible for this large shift in HER onset, we can speculate on two possible explanations: (1) some component of the medium acts as a catalyst, activating the surface of PtIr for HER; (2) the measured H_2_ is not directly the result of cathodic water splitting, but rather electrochemical decomposition of some organic material in the medium, resulting in the release of H_2_ as a byproduct.^34^ The pH changes plotted in Figure 5b show that HER in PBS correlates to alkalization of the solution, while less so in DMEM-FBS. Unbuffered Na_2_SO_4_, in contrast, already becomes alkaline in the ORR region of polarizations around 0 V, due to proton consumption from ORR. Scanning in the anodic direction, it is possible to see acidification in the case of Na_2_SO_4_ and as expected milder acidification in PBS. DMEM+FBS, on the other hand, does not reveal any acidification even up to +1200 mV. Based on the preliminary results of cyclic voltammetry (shown in Figure 2b), it was already apparent that the medium contains easily oxidizable substances. Thus, oxidation of various donor molecules competes with the OER and relatively large anodic currents are supported without any change in pH. In direct measurement of the O_2_ concentrations (Figure 5a), this trend is confirmed. We can establish the onset of OER in PBS at potentials at +800 mV due to the measurable increase in O_2_ (Figure 5a). In unbuffered Na_2_SO_4_, onsets shift to higher potentials due to acidification, pushing the thermodynamic potential to higher values. Meanwhile, the onset of OER is highest in DMEM+FBS, where OER is not clearly present until potentials higher than +1200 mV are applied. Therefore, overall, the “Water window” can be said to span a wider range of potentials in DMEM+FBS versus PBS, though with the caveat that a process different from water electrolysis probably leads to H_2_ evolution at low potentials in DMEM+FBS.

### 3.4 Chloride Oxidation and Platinum Dissolution

**Figure 6:**
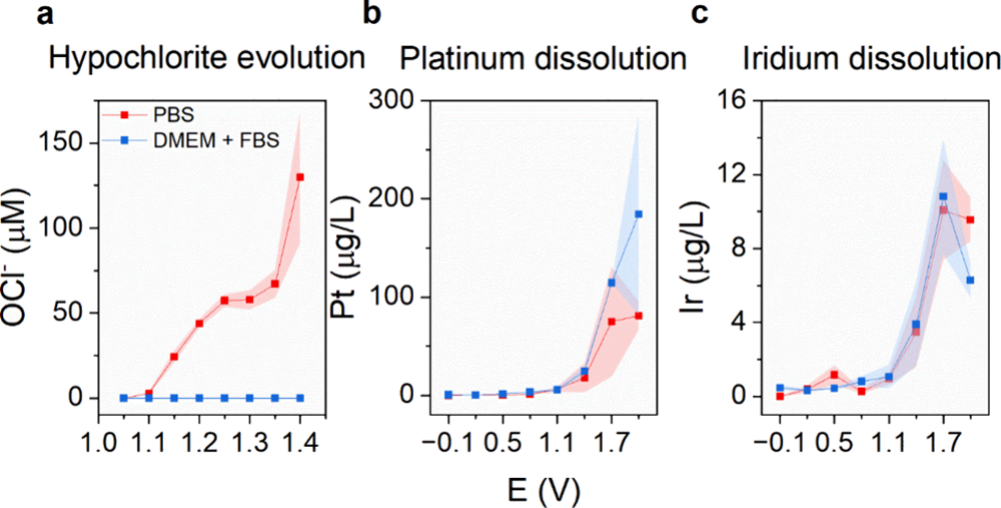
Chloride oxidation and electrode corrosion. (a) Hypochlorite concentration measured via the TMB assay after applying a fixed potential for 5 min in PBS. Hypochlorite was not detectable in the DMEM+FBS medium due to rapid reactivity with the medium. Data for *N* = 3 experimental repetitions, ± SD. (b) Platinum and (c) iridium dissolution measured by ICP-MS from solution aliquots obtained via stepwise application of anodic potentials for 30 min. Pt/Ir *N* = 3 experimental repetitions, ± SD.

Unlike water electrolysis and ORR, chloride oxidation reactions have not been characterized in the neural interface electrode literature. Oxidation of chloride ions results in Cl_2_, which at pH 7 will exist in aqueous solution in equilibrium between hypochlorous acid and hypochlorite (HOCl/OCl^–^). In PBS, the application of potentials greater than +1100 mV results in hypochlorite evolution (Figure 6a). The onset potential of detectable hypochlorite corresponds with what is observed in a comparison of CVs of chloride-free PBS with chloride-containing PBS (Figure 2b). A control measurement in phosphate-buffered Na_2_SO_4_ solution showed that no absorbance signal is produced in the absence of chloride ions, confirming that the TMB-based assay is highly sensitive to chlorine active species. In DMEM+FBS medium, we could not obtain any measurable hypochlorite signal. We hypothesized that similar to the case of peroxide, the produced hypochlorite was rapidly reacting with available organic substrates in the medium. This was confirmed by measuring the stability of hypochlorite in PBS and in the DMEM+FBS medium. The reactivity of hypochlorite added to the medium is so rapid that no signal is produced by the subsequent addition of the assay and measurement of the absorbance (handling of the samples takes roughly 20 s, so within this time, the reaction is complete). Injecting hypochlorite solution directly into a solution of medium with TMB gave a transient blue signal, which faded within seconds. We did not explore the dynamics of this reaction further.

It is known that at anodic potentials high enough for chloride oxidation, platinum will also corrode via the formation of soluble chloride salts (although corrosion in nonchloride electrolytes can occur also).^18,35^ The release of platinum ions from implantable electrodes has been characterized in several studies.^36,37^ The prevalence of corrosion varies with the history of the given platinum electrode. Biphasic application of cathodic/anodic pulses has been reported to lead to more rapid corrosion.^38^ For instance, full reduction of the platinum surface oxide, followed by fixing an anodic potential, will allow the exposed unoxidized surface platinum to more efficiently produce soluble species. We elected to characterize PtIr electrode corrosion with a relatively conservative protocol, involving initial full reduction of the electrode (only at the start of the experimental sequence) and then the stepwise chronoamperometry experiment. Anodic potential steps were applied without intervening cathodic pulses, which would aggravate corrosion more. After each step, the Pt and Ir concentrations were quantified via IC-PMS, after prior calibration via the appropriate standards. The concentrations as a function of applied potential are presented in Figure 6b,c. Trace amounts of Pt and Ir ions are detected starting from a potential of 0 mV, but rising exponentially as higher potentials are applied above +1 V. At these potentials, concurrent OER causes the formation of oxygen bubbles on the electrode surface, which creates inhomogeneities in current (this likely leads to the larger error bars for Pt/Ir at the most positive potentials). The ratio of Pt/Ir ions detected is consistently a factor of 10, corresponding to the known composition of the electrode. This indicates that Pt and Ir corrode from the electrode at equal rates. The presence of proteins has been reported to have a protective effect on the platinum and to decrease the rate of corrosion.^18^ We did not observe this effect. As can be seen in Figure 6b,c, we did not find any differences in Pt or Ir content removed from the electrode when comparing PBS with the DMEM medium. Platinum dissolution has been suggested to be a potential harmful factor in implanted neurostimulators.^36,37^ Soluble chloride complexes of Pt are known to be cytotoxic, most famously cisplatin, an anticancer compound that can be formed by electrochemical oxidation of platinum.

## 4 Conclusions

While Pt and PtIr are extremely well-studied electrodes in the electrochemistry field, the understanding of their electrochemical behavior in the context of bioelectronics and neural interface electrodes is incomplete. The most basic electrochemical reactions, such as water splitting, oxygen reduction, corrosion, etc., are all important considerations in any bioelectronics application. Especially, stimulation electrodes used for *in vitro* or *in vivo* experiments are meant to pass relatively high current densities, and it is well-known that exceeding a certain voltage window can lead to irreversible charge-transfer, also known as faradaic, reactions. Considerations of safety and stability of neurostimulation protocols is a topic of extensive research in the neural engineering field, and many techniques exist to maintain charge balance and minimize DC voltage build-up during stimulation.^10,11^ In parallel, there is an emerging interest in exploring how faradaic reactions may influence physiology beyond just the deterministic neural stimulation impulse.^20,39^ Many molecules vital to signaling and cell homeostasis are redox active, and they can be influenced by neural stimulation electrodes. This may be important not only in understanding possible side effects of neurostimulation but also in harnessing direct pharmacogenomics to lead to desired outcomes. Though many studies in the neural engineering community have focused on PtIr electrode safety and stability, the water window, and the oxygen reduction reaction, the studies are often narrow. What we have set up differently in this study is to (1) use techniques to directly quantify the products of electrochemical reactions (O_2_, H_2_O_2_, H_2_, etc.), instead of just interpreting current/voltage transient data; (2) compare the “standard” PBS solution used by many researchers with cell culture medium, finding that results vary greatly; and (3) reveal a major contribution of chloride oxidation and generation of reactive chlorine, a powerful oxidant. Chlorine has received almost no attention in neural engineering, to the best of our knowledge. Our work suggests an alternative to prevailing methodology to precisely define onset potentials of faradaic reactions: combining “standard” electrochemical techniques such as voltammetry or amperometry can be supplemented by recording concentrations of reactants and products to provide a fuller picture of what is occurring in the vicinity of the electrode surface. To conclude, we remark about the significance of the different reactions we have mapped and comment on possible use cases.

### 4.1 Oxygen Reduction (ORR)

Dissolved oxygen exists under physiological conditions at concentrations in the range of 10–250 μM. *In vitro* experiments with good access to air will have concentrations close to 250 μM. While oxygen is bound to hemoglobin in the bloodstream after being released by red blood cells, oxygen travels from the capillaries to surrounding cells by diffusion. Oxygen is a relatively strong electron acceptor. The reduction potential is +1.23 V more positive than hydrogen evolution; therefore, the reduction of oxygen is highly favored thermodynamically over cathodic water splitting. We have shown that ORR occurs at potentials more negative than +300 mV versus Ag/AgCl. Application of constant cathodic potential to PtIr leads to oxygen reduction and therefore its depletion near the electrode surface. It is possible to electrochemically deoxygenate a region of electrolyte up to several hundred microns away from the PtIr surface. The factor of possibly lowering the oxygen concentration near the electrode should be considered as something that can affect cell function. We would suggest that targeted DC electrochemical deoxygenation, creating a gradient of hypoxia in solution, can be a powerful research tool. Indeed, this concept was recently published.^40^ A final consideration in ORR is that the ORR can proceed via a two-electron pathway, leading to H_2_O_2_ as a byproduct. This effect happens in a “sweet spot” range of potentials. H_2_O_2_ concentrations in the range of tens of micromolar are observed near PtIr electrodes, which are likely not cytotoxic, but are well in the range of action as a signaling molecule.^41^ Low concentrations of peroxide serve as a messenger molecule in various cellular pathways and can affect the gating of certain types of ion channels. Again, this invites the consideration of peroxide produced by ORR as a possible confounding side effect as well as a research tool.

### 4.2 Hydrogen Evolution Reaction (HER)

Cathodic polarizations more negative than those driving the ORR begin to split water and evolve H_2_. In our work, we have tracked this process by applying a H_2_ microsensor to probe the dissolved H_2_, as well as a pH microelectrode, to track alkalization that accompanies HER. These measurements allow the unambiguous assignment of reductive current to cathodic water splitting. An HER onset around −600 mV is consistent with what is expected from the literature on platinum at neutral pH.^28,42^ We were surprised to find a significantly lower onset potential for H_2_ in media, relative to PBS. This signals that some redox process facilitates the evolution of hydrogen, which merits further investigation. The pH changes associated with HER are substantial, despite the buffering capacity of PBS or media, within a few minutes of electrolysis, pH local to the electrode will rise to values >10.

### 4.3 Oxygen Evolution Reaction (OER)

Platinum is a well-known catalyst for the OER. Despite the presence of an anodic oxide on the surface, Pt remains one of the best electrocatalysts for this reaction. In PBS, the onset of OER can be assigned to +800 mV, evidenced by measurable evolved O_2_ local to the electrode. In the medium, the onset shifts to much higher potentials, with no measurable O_2_ evolution until +1100 mV, and overall lower amounts of detectable O_2_ relative to PBS. This is due to the availability of antioxidant species in the medium, which will be readily oxidized more favorably than water. This is clearly apparent by the observation of anodic currents throughout the anodic polarization range being higher than in PBS, indicating that various species are being oxidized. Due to the competitive process of antioxidants being oxidized instead of water, overall OER is less apparent in the medium versus PBS, and also no pH changes are registered. It is worth considering that the medium constitutes a source of “electron donors”, so that if direct current is passed between cathode and anode in biological environment, cathodic reactions like ORR and HER and the associated pH changes may be extensive, while the anodic current completing the circuit simply consumes available organic substrates.

### 4.4 Chloride Oxidation and Electrode Corrosion

The chloride oxidation reaction is rarely considered, despite the fact that all physiological and biological electrolytes contain high chloride concentrations (typically 0.1–0.15 mM). On the PtIr electrode, we find that chloride oxidation competes with the OER and corrosion of Pt and Ir. Clearly measurable concentrations of hypochlorite are detected at potentials more positive than 1.1 V, concurrently with corroded Pt and Ir ions, the detected concentration of which rises exponentially above 1 V. The possibility to generate hypochlorite concentrations of 10–100 μM is intriguing, as these are cytotoxic concentrations, which can be interesting for the ablation of cells or tissues.^25^

Overall, the window of potentials where no faradaic charge-transfer reactions occur on PtIr in a biological medium probably does not exist. The range of +300–600 mV represents the window between the onset of ORR and the onset of OER. This narrow range is the *most* passive, yet at these potentials, some sustained anodic current is still present due to oxidation of organic compounds in the medium. When considering a safety window, an important question in terms of cathodic polarizations will be how sensitive a given biological preparation is to hypoxia, as potentials in the range from −200 to −600 mV lead to deoxygenation of PBS or media with equal efficiency. From the point of view of generating ROS via ORR, PtIr produces relatively low quantities of hydrogen peroxide relative to other electrode materials.^22^ On the anodic side, it must be said that operation in the medium suppresses the effects of the OER and associated pH changes; however, at potentials more positive than +800 mV, the electrode begins to corrode with accompanying release of Pt and Ir ions, and potentials in excess of 1.1 V lead to potentially toxic levels of reactive chlorine and Pt/Ir species.
